# Feeling Trapped and Optimistic: Current Rather than Prospective Medical Conditions Dominate Self-Reported Emotions and Appraisals in Mechanically Ventilated Spinal Cord Injury Patients

**DOI:** 10.1007/s10880-025-10065-5

**Published:** 2025-03-13

**Authors:** Christina Weckwerth, Robert Gaschler, Uwe Hamsen, Aileen Spieckermann, Thomas Armin Schildhauer, Oliver Cruciger, Christian Waydhas, Christopher Ull

**Affiliations:** 1 Faculty of Psychology, FernUniversität of Hagen, Universitätsstraße 47, 58097 Hagen, Germany; 2 Department of General and Trauma Surgery, BG University Hospital Bergmannsheil, Bürkle-de-La-Camp-Platz 1, 44789 Bochum, Germany; 3 Medical Faculty University Duisburg-Essen, Hufelandstraße 55, 45147 Essen, Germany

**Keywords:** Eyetracking, Emotions, Intensive care unit, Self-reported emotions, Spinal cord injury

## Abstract

Adverse medical conditions can involve present and expected future restrictions as a double burden: mechanically ventilated patients with spinal cord injury (SCI), on the one hand, face pain and communication restrictions. On the other hand, they are confronted with significant changes in their future life perspective. While past research on emotion and appraisals has studied SCI patients alone or in comparison with healthy controls, the current work disentangles the potential impact of (a) the adverse current state and (b) expected future restrictions by comparing mechanically ventilated intensive care unit (ICU) patients with vs. without SCI in eye-tracking-based self-reports on emotions and appraisals. Results suggest that patients of either group could provide faceted accounts of their current state, such as feeling trapped and insecure. However, the feedback that SCI and other ICU patients gave was similar, suggesting that current adversities dominate self-reports.

## Introduction

Spinal cord injury has been reported to strongly affect emotions of the affected patients when evaluating the current state as well as the future (Kennedy et al., 2009). Past research has reported high variability in anxiety and depression (e.g., Woolrich et al., 2006) and coping in SCI patients (Kennedy & Rogers, 2000; Kennedy et al., 2009). Furthermore, it has been documented that SCI patients take their life stage, roles, and relationships strongly into account when thinking about their impairment (Kaiser & Kennedy, 2011).

According to Lucas (2007a), individuals who acquire a severe disability report life satisfaction levels that are more than a total standard deviation below their baseline levels, and these levels do not appear to rebound over time. Early on, research stressed that people can sustain at least some happiness despite a spinal cord injury. For instance, participants with spinal cord injuries in Brickman et al. (1978) were above neutral on the happiness scale. In contrast, Lucas (2007a) pointed out that individuals with a SCI were more than three-quarters of a standard deviation below the control group's (individuals without significant impairments or major life events) mean, implying that the average control group participant reported a higher life satisfaction than approximately 78% of participants with spinal cord injuries. In line with these above mentioned results, Dijkers (1997) reported large differences between individuals with spinal cord injuries and healthy participants across studies.

Two issues regarding the emotions of SCI patients are still open and are to be examined in the current research: (1) the point in time and mode of assessment and (2) the group of comparison. Concerning the first issue, past research has mainly collected reports from patients with a long temporal delay after the traumatic event and has focused on patients who could provide self-report verbally or by gestures. This restriction limits our knowledge about patients’ perspectives and needs, particularly during the acute phase in the first weeks after the trauma when patients are awake but not able to communicate verbally due to artificial airways (e.g., tracheostomy and mechanical ventilation). Furthermore, this restriction reduces the options for considering patients’ perspectives during medical treatment. Investigating appraisals earlier and including participants who cannot speak might improve treatment and long-term outcomes. Three factors are mentioned that can help patients endure their stay with minimized risk of subsequent harm: (1) minimal sedation, (2) early sedation, and (3) good communication (Vincent, 2019). Disrupted communication during the stay in the intensive care unit also manifests six months after the patients' discharge and later, in the form of a reduced quality of life (Granja et al., 2005; Tembo et al., 2015). Tackling the difficulties involved with blocked speech due to invasive ventilation and allowing communication about pain and additional emotional strain early on in the ICU might improve the current state as well as long-term prospects. An improved understanding of SCI patients’ feelings and appraisals is necessary, allowing more adequate care while still in the ICU. Eyetracking (ET) can offer a means of communication despite invasive ventilation. ET has proven to be an effective and accurate communication tool in many areas. In the medical-clinical field, ET is used, among other things, as a marker for cognitive disorders (Liu et al., 2021). It should be noted that the effectiveness and accuracy of ET in the intensive care unit have so far only been investigated and demonstrated by a few research groups (e.g., Bodet-Contentin et al., 2022; Ull et al., 2020, 2021, 2022). In prior studies by Ull et al. (2020, 2022)) mechanically ventilated patients, including patients with high paraplegia, were asked about their basic needs (classified into *biological needs* like hunger, thirst, or sleep) and *psychological needs* (experiencing relatedness, experiencing competence, experiencing autonomy) via ET. Assessing the needs of patients during acute treatment in the ICU might reduce or prevent pain and other emotional strain due to SCI and invasive ventilation. For instance, van Diemen et al. (2017) were able to demonstrate that patients with SCI described their bodies as alienated at the beginning of rehabilitation. Tubig and Gilbert (2023) delve deeper into the issue and explain the profound impact that the loss of one’s identity can have, particularly in the context of brain–computer interactions. They highlight that feelings of alienation, loss of self-perception, and changes in body image are common among patients with SCI, with this identity loss often being triggered by the injury itself. Furthermore, they discuss how the explantation of such implantable technologies, such as brain–computer interfaces, can lead to significant changes in personality, identity, autonomy, authenticity, and the self (PIAAAS). This explantation can be a retraumatizing event for those affected, leading to further destabilization of their self-image, underscoring the profound ethical implications associated with both the implantation and explantation of such technologies. Body image explains a large proportion of variance in depression and anxiety disorders, so improving the perception of the body should be a significant part of the rehabilitation process after a spinal cord injury (van Diemen et al., 2017).

Concerning the second issue, past research has either studied SCI patients without a group comparison or compared them to healthy controls. However, this comparison is rather unspecific. Different from other patients with severe medical conditions, spinal cord injury patients might not only be aware of the fact that they have a severe medical condition but also expect the spinal cord injury to be a lasting affliction. So far, there are no data to compare how SCI, compared to other ICU patients, view their future. Past studies have exclusively targeted SCI patients and indicate ambivalence between adaptation processes and the wish for complete recovery. For instance, according to Krause and Edles (2014), 51.6% of SCI patients agreed that “SCI is a serious condition that has life consequences.” However, notwithstanding this evaluation, a majority of patients were optimistic. Only 25.1% agreed to the statement that their SCI is likely to be permanent. Furthermore, optimism concerning long-term developments was widespread, with 86.9% reporting they maintain hope for full recovery, which positively related to reported life satisfaction.

When comparing spinal cord injury patients with healthy controls, it might be challenging to disentangle the potential impact of a severe medical condition from the potential impact on aspects of life satisfaction and well-being. Therefore, we aimed to compare ICU patients with and without spinal cord injury to improve the evaluation of the contribution a patient’s subjective prospects might have on their well-being. In this study, we specifically compared variables related to life satisfaction and well-being among ICU patients with spinal cord injury vs. without spinal cord injury. We hypothesized that spinal cord injury patients might exhibit different levels of life satisfaction and well-being due to their condition, independent of the general severity of their current medical state. The sample was a quasi-randomized sample. We approached all mechanically ventilated patients (with and without SCI) in the ICU units to participate in our study, aiming to include a diverse and representative sample of the ICU population.

## Method

### Sample

The study occurred from February 1st, 2020, to November 30th, 2020, in three intensive care units (medical and surgical intensive care units) at the University Hospital Bergmannsheil Bochum, Germany. One focus of the hospital is the treatment of spinal cord injuries. The following inclusion criteria applied for participation in this prospective study: (1) endotracheal tube or tracheostomy tube and mechanically ventilated, (2) > 18 years of age, (3) mechanically ventilated > 48 h, (4) continued mechanical ventilation for at least 24 h, (5) ability to communicate via non-technical AAC (augmentative and alternative communication; e.g., blink or lip-reading). Any patient who did not fulfill the above inclusion criteria and/or had a speaking ability of 6 h or more was excluded from the study. Patients were also excluded if they experienced excessive agitation, sedation, or delirium as indicated by a score other than − 1, 0, or 1 on the RASS (Richmond Agitation Sedation Score) or a score > 3 on the NU-DESC (Nursing Delirium Screening Scale) in patients without sedation. Informed consent to participate was obtained from all patients. Patients could consent to participate by nodding their heads or blinking. If patients were unable to nod or blink to provide consent, a legal representative provided consent for their participation in the study. A specialist in trauma surgery and a psychologist supervised the entire study.

### Statistical Analysis

An a-priori power analysis was done with G*Power® Version 3.1 (Faul et al., 2009). It showed a sample size of 2 × *n* = 36 for a power of 0.80 with an *α*—error of 0.05 and an effect size of *d* = 0.6. Ninety-five mechanically ventilated and intubated patients fulfilled the inclusion criteria, and 20 patients had to be excluded because of cognitive impairment (*n* = 8), extubation before accomplishment of the testing (*n* = 6), language barrier (*n* = 3), refusal (*n* = 2), or sudden death (*n* = 1). Consequently, 75 patients participated in the study (*M*_age_ = 53, SD = 17.8), of which 21.3% were female, and 78.7% were male (further demographic data see Table 1 in the Appendix).

For analysis, the sample was divided into two groups: spinal cord-injured (SCI; *n* = 46) and non-SCI (*n* = 29) patients. Non-SCI diagnoses comprised COPD (chronic obstructive pulmonary disease), heart failure, or acute abdomen.

To analyze the collected data, various statistical methods were employed. Descriptive statistics were used to summarize demographic data, a t-test for independent samples was utilized to compare the responses of SCI and non-SCI patients on several variables measured by the Visual Analog Self-Esteem Scale (VASES). This test was specifically applied to examine whether SCI patients provided more negative responses compared to non-SCI patients. In addition, the t-test was used to compare specific items such as feelings of being trapped and levels of optimism between the two groups. To further investigate differences in categorical data, particularly concerning future concerns and the perceived impact of the eye-tracking device on quality of life, a chi-square (*χ*^2^) test was conducted. This test assessed the frequency distribution of affirmative responses between SCI and non-SCI patients, allowing for the identification of significant differences in response behavior.

Statistical analysis was performed using Microsoft Excel and IBM SPSS Statistics Version 27.

### Eyetracking

To allow differentiated communication with these non-verbal patients, we used the same eye-tracking technology (ET) used in previous studies (Ull et al., 2020), the Tobii Dynavox I-15 + system (Tobii Dynavox, Danderyd, Sweden). The system includes commercial Windows 10 software and an integrated infrared camera, illumination, and processing units. The integrated camera and video-analysis algorithms were used to determine the gaze point, which was used like a mouse pointer. The patient could now *click* on a possible answer presented on the screen using their gaze. The ET was attached to a movable and height-adjustable arm with wheels to be positioned close to the bed and in front of the patient. The participants were introduced to the Tobii Dynavox for the first time as part of this study, so a training session was implemented prior to data collection. The training that the patients received and the method used to confirm their understanding of the instructions were previously described in the pilot study conducted by our research group (Ull et al., 2020). All patients were introduced to the use of the ET device by playing a simple puzzle game for approximately 10 min to get acquainted with the technique. After completing this short learning phase, the participants proceeded to the main examination phase. The first step involved calibrating the ET device with each patient before each session. Using a 9-point calibration method on the monitor. This ensured that the device accurately tracked the patients' gaze. Following the successful training and calibration phase, the questions were presented to the participants.

In addition to the images, audio feedback was provided for each question and response option. No time limit for answering the question was specified. The patient was forwarded to the next question if their gaze remained on the selected answer option for at least 1000 ms, and the corresponding audio feedback was heard. Furthermore, a back/forward button provided the possibility to return to the previous question or to proceed to the next question.

### Self-Report Instruments

The answers of SCI patients and other ventilated patients were compared for the current study. Some self-report instruments can be applied to patients who cannot speak or communicate by gestures. The visual analog self-esteem scale (VASES) was developed to assess mood, anxiety, depression-tendency, and self-esteem in patients whose use of language is limited (Brumfitt, 1999). The scale offers dichotomies concerning feelings and appraisals (seeking contact vs. not seeking contact; pessimistic vs. optimistic; clear vs. chaotic thoughts; dull vs. sharp thoughts; cheerful vs. not cheerful; angry vs. relaxed, not understood vs. understood; frustrated vs. satisfied; sad vs. happy; insecure vs. secure; trapped vs. free) that ICU patients can use to categorize their state and perspective. In the VASES, pairs of images are shown to the patient. A written description is presented above each picture. The patient's answers are determined by choosing one of the options below the picture (++, +) or the neutral answer option (0) between the pairs of pictures. We chose the VASES for our study because it uses images that are easy to understand and interpret, making it accessible for patients with language impairments and those physically unable to complete a questionnaire independently due to their SCI. The scale is brief yet meaningful, and the 'feeling trapped' item likely reflects a sense of physical entrapment, which is relevant for patients experiencing the dual burden of spinal cord injury and mechanical ventilation.

In addition to the VASES (e.g., reporting feeling trapped), we focused on questions reflecting on psychological needs (experiencing relatedness, experiencing competence, experiencing autonomy) and on basic needs related to current and future concerns (with a binary response format): (1) Do you think a lot about the future?, (2) Are you afraid of losing your job?, (3) Are you afraid of being unable to pursue hobbies?, (4) Are you afraid of needing constant help?, (5) Are you afraid of being alone due to your disease?, (6) Are you afraid your partner could leave you?, (7) Do you think your health will improve? (8) Do you think you could get used to the current state of health? (9) Do you think you will need permanent mechanical ventilation? (10) Do you think this device will improve your quality of life?

## Results

**Fig. 1:**
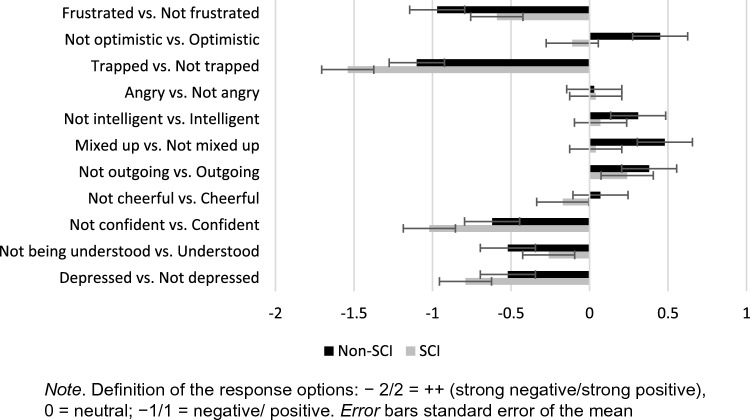
Mean values for the VASES items throughout all patients

First, on the VASES, a t-test for independent samples was used to check whether SCI patients gave more negative responses than non-SCI patients. This was not the case. The SCI patients (*M* = − 0.37, SD = 0.40) did not differ from the non-SCI group (*M* = − 0.18, *SD* = 0.49, *t*(73) = 1.843, *p* = 0.069). However, as shown in Fig. 1, there were substantial mean differences among VASES items. For instance, reports of feeling trapped received a high average rating (in both groups of patients), while on average, there was no tendency to report angriness. The pronounced difference in average ratings across items underlines that patients could read and understand the items and express their answers via gaze point.

While SCI patients were not generally more negative in their reports on the VASES (see above), it needs to be taken into account that one of the items might specifically address the situation of SCI patients compared to non-SCI patients. Accordingly, as a follow-up, we tested for group differences in reports of feeling trapped. Patients with spinal cord injury (*M* = − 1.54, SD = 0.50) felt significantly more trapped than other patients (*M* = − 1.10, SD = 1.15; *t*(73) = 2.286, *p* = 0.025). Furthermore, one VASES item addressed optimism and was used for item-based group comparison. The Spinal cord injury group (*M* = − 0.11, SD = 0.97) was lower in optimism compared to the non-SCI patients (*M* = 0.45, SD = 1.06; *t*(73) = 2.339, *p* = 0.022). For the remaining VASES items, there are no significant differences between the groups (Table 3 in the appendix).

While most VASES items lead to responses on the negative side, some were, on average, neutral or slightly positive. On the one hand, the patients suffering from a severe injury needing permanent mechanical ventilation reported high frustration and a tendency towards depression. On the other hand, many still show optimism.

Next, we compared reports of SCI- and non-SCI patients on current and future concerns related to basic needs (see Table 2). In a binary format, five items asked participants whether they feared specific restrictions in their lives. We computed the percentage of affirmative responses per participant group. The independent samples t-test showed that patients with spinal cord injury (*M* = 53%, SD = 31%) were not significantly more anxious about their future than patients from the non-SCI group (*M* = 41%, SD = 35%, *t*(73) = − 1.443, *p* = 0.153).

Similar to the VASES, the reports on current and future concerns related to basic needs show a differentiated profile (high affirmation rates for both groups on some items and low affirmation rates for both groups on other items). Again, this underlines that participants could provide a differentiated self-report using the eye-tracking setup. Only one item showed a significant difference between the two groups: *Do you think this device will improve your quality of life?* This question referred to the possible impact of eye-tracking-based communication: 78.9% of the SCI patients answered affirmatively, while only 50% of the non-SCI patients did (*χ*^2^(1) = 5.87, *p* = 0.015).

Table 2 shows the results of the contingency table response frequencies in percent for the questions answered “yes” and the chi-square test results detecting differences in response behavior concerning the future.

## Discussion

The current study was the first to compare self-reports on emotions and appraisals among mechanically ventilated patients with vs. without spinal cord injury. ET-based self-report was used to overcome restrictions regarding sample, measurement timing, and group of comparison that had limited earlier studies. For instance, when comparing spinal cord injury patients with healthy controls, it had been difficult to disentangle the potential impact of a severe medical condition from the potential impact of problematic prospects on life satisfaction and well-being. The current study provides a comparison disentangling these possible factors. Results suggest that patients of either group could provide faceted accounts of their current state, such as feeling trapped and insecure. While emotions and appraisals showed a differentiated profile concerning the different items, this profile, in general, was primarily similar for SCI and other ICU patients, suggesting that current adversities dominate self-reports. The SCI- and non-SCI patients of the current study were alike regarding being treated at an ICU for severe medical conditions while being mechanically ventilated and unable to communicate verbally. There were specific differences due to the particular condition of SCI patients. SCI patients more strongly reported feeling trapped and, at the same time, more frequently underlined the value of eye-tracking to enable communication and, by this, improve life quality. Non-SCI patients reported more optimism than SCI patients. The latter were more open to getting used to their current state of health.

In the current study, we used eye-tracking to acquire self-reports on emotions and appraisals of mechanically ventilated patients with and without SCI. It needs to be clarified whether mechanically ventilated patients with vs. without SCI differ in emotions, appraisals, and outlook. Until recently, communication restrictions have drastically reduced the possibility of taking the subjective needs of these patients into account. However, apart from restricted options for discovering what patients view as their burdens, restricted communication—in and of itself—seems to be one of the major burdens from the perspective of the patients. Invasively ventilated patients in the intensive care unit experience voicelessness as particularly frustrating (Yang, 2016). Some described communication impairment as one of the most stressful, dehumanizing, and frustrating aspects of their ICU stay (Baumgarten & Poulsen, 2015; Guttormson et al., 2015; Karlsson et al., 2012; Patak et al., 2006). According to Happ et al. (2015), over 50% of ICU patients are alert enough to communicate with their caregivers and attending physicians. However, they may not be understood because of communication impairments from an artificial airway. Interactions between patients and medical teams usually focus on the content perceived as relevant by clinicians but less on the content perceived as necessary by patients (Foster, 2010; Leung et al., 2018).

Given that mechanically ventilated ICU patients with or without SCI share this burden of impaired communication, their emotions and appraisals might be similarly dominated by these acute restrictions, leading to similar reports. Our findings support this notion, as we observed that patients with SCI tend to base their appraisals and emotions predominantly on their current circumstances, shaped by the acute restrictions of ICU treatment, rather than future-oriented considerations. This potential dominance of current adversities in reports on emotion and appraisal is in line with the theory of socio-emotional selectivity (Carstensen, 1991; Carstensen et al., 1999). The theory states that people shift their motivation over their lifespan, driven by their subjective future perspective rather than chronological age. Positive emotions and important social partners are highly valued when the remaining life span is perceived as limited. This has, for instance, been reported by people suffering from HIV. Likely people with a high degree of paraplegia who are permanently dependent on mechanical ventilation also recognize restrictions in future perspective compared to circumstances not dominated by ICU treatment. The overall mortality of ventilated patients is 35%, of which approximately 32% die within one year (Hirschfeld, 2020). Our findings are in line with this, suggesting that these immediate challenges may intensify the focus on present conditions in shaping emotions and appraisals. Patients might sustain some positive emotions and appraisals by considering current supporting factors, such as alleviating communication restrictions by the ET-based setup, medical treatment reducing pain, and support from the social environment while in the hospital. In this context, our study highlights the importance of mitigating current adversities, as we observed that improving communication through the ET-based setup not only alleviates frustration but also contributes to sustaining positive emotions.

According to Bach and Tilton (1994), people with complete traumatic tetraplegia reported life satisfaction at levels nearly equal to or even exceeding the level reported by people with no health impairment. Schwartz et al. (2018) suggested as an explanation that persons with spinal cord injury have presumably adapted to their circumstances and that standard measurement instruments do not capture response shift effects. Our findings support this perspective, as patients with spinal cord injury (SCI) might evaluate their emotions and quality of life based on their immediate circumstances, which could reflect the possible activation of cognitive and emotional adjustments. Response shift effects are observed in individuals whose health status changes drastically (Sprangers & Schwartz, 1999). Response shift theory offers an approach to why individuals with an SCI might tend towards optimism. The theory postulates that (1) stable characteristics of an individual (so-called antecedents) interact with (2) cognitive-affective and behavioral strategies (mechanisms) and influence cognitive appraisals that, (3) moderate the effect on one's changed health status on the perceived quality of life (i.e., response shift) (Rapkin et al., 2004; Sprangers & Schwartz, 1999). In line with this, it is possible that patients with SCI tend towards optimistic appraisals of their current situation due to their ability to cognitively reframe challenges and adjust their expectations.

The aspects mentioned above relate to the disability paradox. Why do people with disabilities, including those with SCI, report optimism or good to high life satisfaction? The central point of this paradox lies in a balance of body, mind, and soul and the maintenance thereof, combined with a stable social environment (Albrecht & Devlieger, 1999). Previous studies on basic needs in SCI patients have shown that SCI patients indeed feel embedded in a good social environment (Ull et al., 2022). Seventy-five percent of SCI patients reported feeling understood by their family members, and 66% did not feel abandoned by their family members (Ull et al., 2022). It should be mentioned that the group of SCI also included persons with acute SCI, which meant that the survey took place in a relatively short period after the accident. Similarly, our findings suggest that positive social interactions and support systems might play a critical role in shaping current appraisals of well-being, even shortly after the onset of SCI. However, this raises the question of how quickly adaptation occurs in individuals with an acute SCI, mainly since the response shift effect has been observed in individuals with chronically ill conditions (Finkelstein et al., 2014; Schwartz et al., 2004) and the response shift effect itself is not seen as an adaptation, but rather as an effect of adaptation (Schwartz et al., 2013). According to Schwartz et al. (2018), the response shift effect applies to individuals with chronic SCI. “People appeared to change their internal standard regarding physical functioning and general health over time, …” (Schwartz et al., 2018), so it can be assumed that individuals are responding from the current perspective. Our results might complement this view, as patients with acute SCI already tend to evaluate their situation based on their current perspective, suggesting that adaptation might begin earlier than previously assumed. Since the number of those with an acute SCI is highly represented, it can also be assumed that the adaptation to the given circumstances takes place promptly, and the response shift effect is also valid for people with an acute SCI. However, the acceptance of the current health status is low. The possible adaptation process points towards a hedonistic adjustment in which individuals return to their basic level of happiness after a change in their life circumstances (Lucas, 2007b). Future research should focus on the response shift in acute illness. In this context, it should be examined whether diagnosis-dependent differences exist in how quickly and effectively adaptation occurs, as these differences warrant further investigation.

Previous research has demonstrated a wide range of psychological adjustment in patients with SCI (Galvin & Godfrey, 2001)—Lazarus’ stress, appraisal, and coping model being a dominant reference (Lazarus & Folkman, 1984). The optimistic tendency in some SCI patients may be a form of hope. Hope is described as the expectation to achieve a goal in the future (Tong et al., 2010). In their study, Dorsett et al. (2017) dealt with the construct of hope in SCI patients and found that hope correlates positively with acceptance. The current study, however, points in a different direction—suggesting that hope can be high while acceptance is low. On the one hand, 76.3% of the SCI patients and 80.8% of the non-SCI patients reported that their health would improve. On the other hand, acceptance was low. Only 31.6 and 11.5% of the patients indicated they think they will get used to the current state of health. The results for people with SCI are consistent with those of Krause and Edles (2014), who report that hoping for improved health status predicts life satisfaction in SCI patients. Given the overlap of VASES reports in SCI and non-SCI patients, future work might shift towards other aspects of the current treatment, which might powerfully shape patients` appraisals of their current situation. The ventilation variant used might be such a variable. For instance, Huttmann et al. (2018) screened people with long-term home mechanical ventilation (HMV). They pointed out that their life satisfaction was severely impaired despite maximal patient care and full support of technical aids. However, the current investigation suggests that technical aids for communication can make a difference from the patient's perspective. In our sample, 78.9% of SCI patients and 50% of non-SCI patients reported that using an eye tracker would improve their quality of life. Improved communication can also help address that patients’ future perspectives, in part, seem to be dominated by their current treatment. For instance, while non-SCI participants would likely be weaned from ventilation, almost 40% believed they need permanent ventilation.

## Limitations

Some work regarding the impact of major life events on life satisfaction has used repeated measures before and after the life event, allowing the study of effects over some time and taking individual baseline levels into account (Lucas, 2007a). According to Lucas (2007a), individuals show drops in their level of life satisfaction and optimism of more than one standard deviation after a critical life event (for example, the onset of paraplegia) and often remain on this reduced level. While unable to provide data from before the major life event, we could assess severely affected patients during their ICU stay while facing communication restrictions due to mechanical ventilation. It should be noted that comorbidities might have impacted their biological and psychological needs, but this was not explicitly recorded.

As a further limiting factor, it should be mentioned that the group of spinal cord-injured patients included 15 participants who were not in the ICU because of high paraplegia and the permanent need for ventilation as the primary diagnosis but because of another diagnosis (for example, after visceral surgery). So, for some of the patients, paraplegia was not a novel health restriction. Future research should also consider the distinction between acute and chronic spinal cord injury. Also, concerning ventilation, future studies should differentiate between the (subjective) permanent vs. temporary need for such support in detail. Previous research on coping strategies after paraplegia takes the level of paraplegia or the ASIA (American Spinal Injury Association) classification into account (Dorsett et al., 2017). However, few studies consider whether mechanical ventilation is permanently required. At present, it can be assumed that the subjective estimations of patients with high paraplegia and continuous mechanical ventilation concerning their future are based on their present condition. In addition, it can be assumed that those with an acute SCI might not fully grasp their prognosis.

Some of the patients have been living with the diagnosis of high paraplegia and continuous mechanical ventilation for years. Barone and Waters (2012) describe the influence biopsychosocial factors have on how patients cope with SCI and highlight that relatively recently injured patients are more prone to an avoidance and repression mechanism. However, this is contradicted by findings by Martz et al. (2005), who investigated predictors of psychosocial adjustment to spinal cord injury in a study with 313 spinal cord-injured patients. The severity and duration of the existing injury provided only weak evidence for the assumption that a longer duration, even with a severe degree of spinal cord paralysis, results in a more favorable adjustment. Similarly to the participants in this study, most patients had a severity of injury at level A according to the ASIA Impairment Scale. Grade A of the ASIA Scale is reflected by a complete loss of sensory and motor function below the level of injury.

## Conclusion and Future Perspective

In this study, eye-tracking was used to determine emotions and appraisals of mechanically ventilated SCI and non-SCI patients. The differentiated profiles indicate that gathering information directly from the patients via eye-tracking is highly relevant. The current work suggests that, while being treated in the ICU, reduced life satisfaction and optimism are mainly driven by the severe current health restrictions rather than by the subjective prospects that might differ between SCI and non-SCI patients.

Despite the severity of the injury, the limited scope for possible improvement in health status, and the feeling of being trapped, patients with high paraplegia show optimism. This result is consistent with Brickman et al.'s (1978) findings that ICU patients with and without SCI can maintain some life satisfaction.

ET-based communication in the intensive care unit can benefit patients with permanent ventilation, especially for patients with high paraplegia (Ull et al., 2020). The results of the current study suggest that current adversities dominate emotions and appraisals. It seems reasonable to repeatedly conduct ET-based assessments of emotions, appraisals, and needs, allowing the information and treatment provided to the patient to be adjusted and improved as appropriate for their current state. The VASES, which records moods, anxiety, and depressive tendencies, is particularly suitable for this purpose. It allows psychotherapeutic measures to be taken more quickly if necessary or anxiety-relieving medication to be prescribed if required. Alongside psychotherapeutic measures and anxiety-relieving treatments, it is crucial to consider the social factors that impact patient well-being. Our results indicate that patients primarily experience fears of loss related to their partner, friends, or job, likely due to feelings of diminished competence, autonomy, and social integration. According to the self-determination theory (SDT) (Ryan & Deci, 2017), these factors are essential for perceived well-being. To enhance social connectedness, alternative augmentative communication (AAC) tools, such as ET, are valuable, as demonstrated in our pilot study (Ull et al., 2020). Building on these findings, our ongoing research aims to develop a needs- and patient-centered application using webcam-based eye-tracking for non-communicative and ventilated patients in the ICU, enabling them to interact with their social environment.

## Data Availability

The datasets used and/or analyzed during the current study are available from the corresponding author on reasonable request.

## Appendix

See Appendix Fig. 1 and Tables 1, 2, and 3.

**Table 1:** Demographic data of enrolled patients (n = 75)

	Total
Gender m:f	59 (78.7%): 16 (21.3%)
Age (years)	58.3 ± 17.8
LOS hospital (days)	88.7 ± 81.9
*Reasons for ICU admission*	
Acute spinal cord injury (aSCI)	31 (41.3%)
Chronic spinal cord injury (cSCI)	15 (20.0%)
Days since onset of SCI to survey*	6.8 ± 10.14
Other diagnoses	29 (38.7%)
CCI (points)	2 ± 2
SOFA score (points)	8.8 ± 1.9
Days MV to survey	26.96 ± 20.74

Data presented as absolute numbers (percentage) or mean ± standard deviation

*m* male, *f* female, *LOS* length of stay, *ICU* intensive care unit, *aSCI* newly acquired SCI, *cSCI* readmitted after previous treatment for SCI due to subsequent complications or illnesses, *CCI* charlson comorbidity index, *SOFA* sepsis-related organ failure, *MV* mechanical ventilation

*Refers to patients with acute SCI

**Table 2:** Results of the contingency table for the percentage response frequencies (answered “yes”) and the results of the chi-square test detecting differences in response behavior concerning the future divided into spinal cord injury (SCI) and non-spinal cord (non-SCI)

Items	SCI (%)	Non-SCI (%)	*χ*^2^	*p*
Are you afraid of losing your job	37.0	20.7	2.21	.137
Are you afraid of being unable to pursue hobbies?	54.3	41.4	1.20	.274
Are you afraid of needing constant help?	67.4	65.5	.028	.867
Are you afraid of being alone due to your disease?	56.5	51.7	.165	.684
Are you afraid that your partner could leave you?	47.8	27.6	3.03	.081
Are you thinking a lot about the future?	67.4	48.3	2.71	.100
Do you think your health will improve?	76.3	80.8	.179	.672
Do you think you get used to the current state of health?	31.6	11.5	3.45	.063
Do you think you will need permanent mechanical ventilation?	36.8	38.5	.017	.895
Do you think this device will improve your quality of life?	78.9*	50.0	5.87	.015*

****p* < .05

**Table 3:** Results of the intergroup comparison of VASES Items

Item	Spinal cord injury	Non-spinal cord injury		
	*M* (SD)	*M* (SD)	*t*(73)	*p*
Depressed_Not depressed	− .72 (.88)	− .62 (.82)	− .545	.587
Not cheerful_Cheerful	− .03 (.81)	− .10 (.72)	.424	.673
Not feeling trapped_Feeling trapped	− 1.54 (.50)	− 1.10 (1.15)	2.286	.025*
Not optimistic_Optimistic	− .11 (.97)	.45 (1.06)	2.239	.022*
Not confident_Confident	− .83 (1.18)	− .90 (1.07)	.246	.806
Frustrated_Not frustrated	− .64 (.90)	− .77 (.81)	.660	.511
Mixed up_Not mixed up	.19 (.86)	.23 (.93)	.431	.861
Not being understood_Understood	− .31 (1.12)	− .41 (1.04)	.420	.676
Not outgoing_Outgoing	.31 (.82)	.28 (.79)	.126	.900
Not intelligent_Intelligent	.19 (1.01)	.13 (.73)	.327	.744
Angry_Not angry	− .14 (.93)	.21 (1.01)	− 1.535	.129

**p* < .05
